# Immune Enhancement of Fermented *Ruditapes philippinarum* Polysaccharide on Immunosuppressed *BALB/c* Mice Induced by Cyclophosphamide

**DOI:** 10.3390/molecules30234583

**Published:** 2025-11-28

**Authors:** Ting Zhang, Jiale Song, Zhenzhen Peng, Mengjiao Wu, Zhi Li, Fei Li, Yuxi Wei

**Affiliations:** 1 College of Life Sciences, Qingdao University, Qingdao 266071, China; zhangtlh27@163.com (T.Z.); jlsong2021@163.com (J.S.); zhili1198@163.com (Z.L.); 2 Chongqing Institute for Food and Drug Control, Chongqing 401121, China; 3 Qing Haier Smart Technology R&D Co., Ltd., Qingdao 266000, China; wumengjiao@haier.com

**Keywords:** fermented *Ruditapes philippinarum* polysaccharide, immunomodulatory, intestinal mucosal barrier, immunosuppressive *BALB/c* mice

## Abstract

Polysaccharides from marine organisms have been extensively studied and utilized as functional food ingredients due to their excellent immunomodulatory properties. However, the immunomodulatory potential of fermented *Ruditapes philippinarum* polysaccharide (RPP) has not been systematically explored. This study investigated the effects of RPP on immune function in cyclophosphamide (CTX)-induced immunosuppressed *BALB/c* mice. These results revealed that RPP alleviated CTX-induced weight loss and restored appetite. Moreover, RPP can promote the morphology and indices of immune organs, as well as increased the number of white blood cells (WBC), red blood cells (RBC), and hemoglobin (Hb). Serum levels of interleukin-6 (IL-6) and immunoglobulin E (IgE) were significantly elevated following RPP treatment. Additionally, RPP improved colonic morphology by upregulating the expression of E-cadherin and ZO-1 and promoting the secretion of secretory IgA (sIgA). These results indicated that RPP exerted an immune protective effect in *BALB/c* mice and justified its further potential as a bioactive ingredient for functional foods derived from marine shellfish.

## 1. Introduction

The immune system comprises specialized organs, cells, and soluble mediators that collectively maintain host homeostasis by eliminating invading pathogens [1]. This complex process involves various cell types and organs in recognizing and eliminating foreign microbes and substances. For example, macrophages release cytokines upon identifying a target [2]. Cyclophosphamide (CTX), a powerful alkylating cytostatic, is used to malignancy control. However, it also markedly suppresses host immunity, mainly reflected by organ injury [3,4]. Therefore, to mitigate CTX-induced toxicity while potentiating its antitumor activity, extensive research studies have been directed toward the discovery of immunomodulatory therapeutics. Notably, natural products are extensively used as immunostimulants due to their abundance, higher safety profile, and lower toxicity [5,6].

Increasing evidence indicate that bioactive substances (such as polysaccharides, peptides, glycoproteins and agglutinins, etc.) has become a focal point for investigators seeking treatments for immune function decline in marine shellfish [7,8,9,10]. Among them, marine polysaccharides are naturally ubiquitous macromolecules that exert diverse physiological activities, including antitumor, antioxidant, hypolipidemic, and hypotensive effects [11,12,13]. For instance, the galactans (SP1 and SP2) isolated and extracted from green algae had a stimulating effect on macrophages by inducing the enzyme inducible nitric oxide synthase to lead to the production of NO. SP1 and SP2 can also increase the level of interleukin-6 (IL-6) in the culture supernatant from RAW 264.7 mouse macrophages [7]. Oyster polysaccharides (OP-1) had been demonstrated to exert antitumor effects by promoting splenocyte proliferation and interleukin-2 (IL-2) release while simultaneously inhibiting HepG2 cells proliferation [14]. In addition, Microwave-extracted clam polysaccharide (MCP) not only lowered intracellular reactive oxygen species (ROS) in tumor cells, but also boosted antitumor efficacy via simultaneously activating immune responses and modulating redox balance [15]. In addition, *Ruditapes philippinarum* polysaccharides alleviated hyperglycemia by increasing the concentration of short-chain fatty acids (SCFAs) and improving medium-chain fatty acids (MCFAs) and secondary bile acids (SBA) in type 2 diabetes mellitus (T2DM) mice [16]. Nevertheless, the presented studies on marine bivalve derived polysaccharides have predominantly focused on metabolic diseases or anti-tumor activity. Their efficacy under immunosuppressed conditions, particularly in CTX-induced immunodeficiency, remains largely unexplored.

*Ruditapes philippinarum* (*R. philippinarum*) is a commercially important shellfish species in Chinese aquaculture, widely distributed along the coasts of China, Korea, Japan, and Spain [17,18]. Research studies on *R. philippinarum* have predominantly focused on polypeptides, unsaturated fatty acids, and polysaccharides extracted by non-fermentation method [19,20,21,22]. However, fermentation technology has been shown to modify the structure of polysaccharides, thereby enhancing their physiological activities [23]. In this study, CTX administration established an immune-compromised mice model, followed by evaluation key immune parameters and intestinal barrier indicators of fermented *Ruditapes philippinarum* polysaccharide (RPP). The findings confirmed the immunomodulatory efficacy of RPP and supported the progression of clinical development of RPP as an immunomodulatory agent.

## 2. Results

### 2.1. Effects of RPP on Body Weight and Food Intake in BALB/c Mice

**Figure 1:**
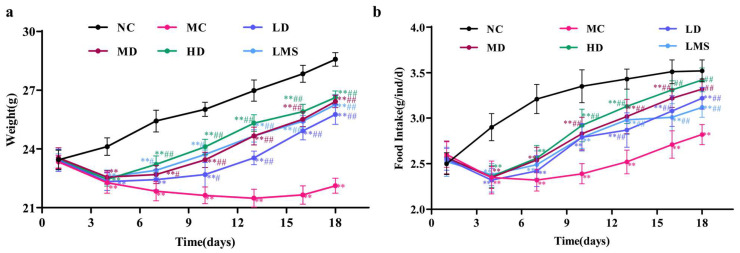
The effect of RPP on the weight change and food intake of mice (n = 6). (**a**) Changes in body weight of immunosuppressed *BALB/c* mice; (**b**) Changes in food intake of immunosuppressed *BALB/c* mice. Note: ** *p* < 0.01, extremely significantly different from the NC group; ^#^ *p* < 0.05, significantly different from the MC group; ^##^ *p* < 0.01, extremely significantly different from the MC group. Data are shown as mean ± SD.

Mice with CTX-induced immunosuppression exhibited marked reductions in both body weight and food intake [24]. As shown in Figure 1a, body weights did not differ markedly among groups before model establishment (*p* > 0.05). After CTX administration, the model (MC) group showed a marked reduction in body weight relative to the control (NC) group (*p* < 0.01), confirming successful induction of immunosuppression. The RPP groups exhibited significantly higher body weights than the MC group (*p* < 0.05 or *p* < 0.01), demonstrating that RPP effectively mitigated CTX-induced weight loss in immunosuppressed *BALB/c* mice. Specifically, the body weights of the low-dose (LD), medium-dose (MD), and high-dose (HD) groups were 3.64 g, 4.31 g, and 4.51 g higher than that of the MC group (22.12 g), respectively. Moreover, the HD group was more effective at mitigating weight loss than the levamisole hydrochloride (LMS) group.

Changes in food intake were strongly correlated with trends in body weight (Figure 1b). CTX administration significantly reduced food consumption in the MC group versus the NC group (*p* < 0.01), confirming the successful establishment of the immunosuppression model. RPP administration improved food intake in a dose dependent manner. Compared to the MC group, RPP-treated groups showed increases of 0.40 g, 0.50 g, and 0.60 g, respectively (*p* < 0.01). Notably, RPP-treated groups exhibited more pronounced therapeutic effects in restoring food intake compared with the LMS group. All in all, RPP effectively reversed the body weight loss and the reduction in food intake caused by CTX in *BALB/c* mice.

### 2.2. Biosafety Assessment of RPP

The toxicological assessment of RPP can be assessed by calculating organ indices (heart, lungs, liver, and kidneys) in mice [25]. As shown in Table 1, main organ indices of the RPP-treated groups demonstrated no marked difference compared to those of the NC group (*p* > 0.05), suggesting that RPP had no obvious toxicity to vital organs at the tested dosage. However, the lung index in the MC group (9.18 mg/g) showed a significant increase compared to the NC group (7.24 mg/g) (*p* < 0.05). This finding was consistent with previous research results and also demonstrated that CTX increased the lung index, which may be related to the fact that CTX causes pulmonary edema [26].

### 2.3. Effects of RPP on Immune Organ Indices

**Figure 2:**
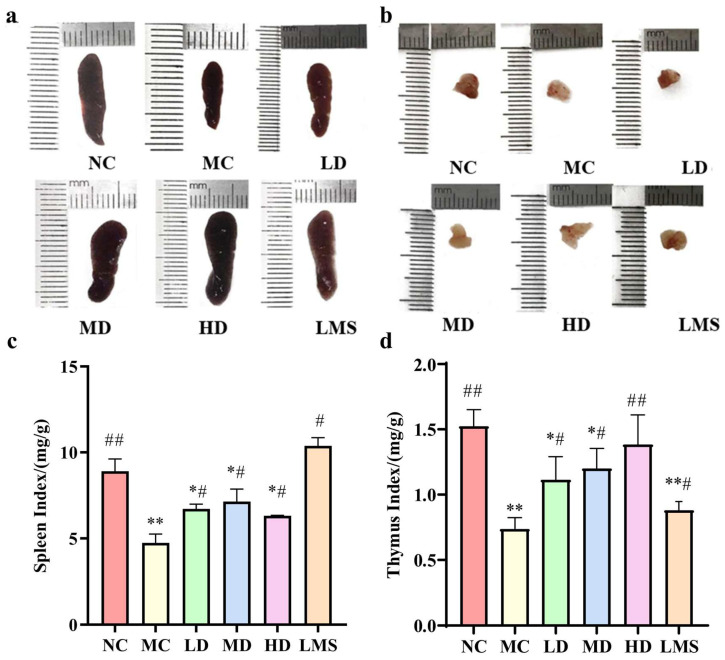
Effects of RPP on the immune organs of immunosuppressed *BALB/c* mice (n = 6). (**a**) The effect of RPP on the morphology of the spleen of immunosuppressed *BALB/c* mice; (**b**) The effect of RPP on the morphology of the thymus of immunosuppressed *BALB/c* mice; (**c**) The effect of RPP on the spleen index of immunosuppressed *BALB/c* mice; (**d**) The effect of RPP on the thymus index of immunosuppressed *BALB/c* mice. Note: * *p* < 0.05, significantly different from the NC group; ** *p* < 0.01, extremely significantly different from the NC group; ^#^ *p* < 0.05, significantly different from the MC group; ^##^ *p* < 0.01, extremely significantly different from the MC group. Data are shown as mean ± SD.

Spleen and thymus are pivotal components of the immune system, playing a crucial role in orchestrating the body’s defense mechanisms. Moreover, the indices of these immune organs can provide a reliable parameter for evaluating immune function in CTX-induced immunosuppressed *BALB/c* mice [19]. As depicted in Figure 2, the MC group demonstrated significant atrophy and a notable reduction in the organ indices of both the spleen and thymus compared to the NC group (*p* < 0.05). These observations collectively confirmed the successful establishment of the immunosuppressive model. Compared with the MC group, RPP significantly reversed CTX-induced atrophy of immune organs and reflected by the recovery of their organ indices (*p* < 0.05). In particular, the thymus index of the HD group was extremely significantly higher than that of the MC group (*p* < 0.01), reaching levels comparable to those in the NC group (*p* > 0.05). Notably, the therapeutic effects of RPP-treated groups on the thymus were superior to that of the LMS group. These findings revealed that RPP possessed the capability to enhance the indices of immune organs, with a notably pronounced protective efficacy on the thymus.

### 2.4. Effects of RPP on Blood Routine Parameter

**Figure 3:**
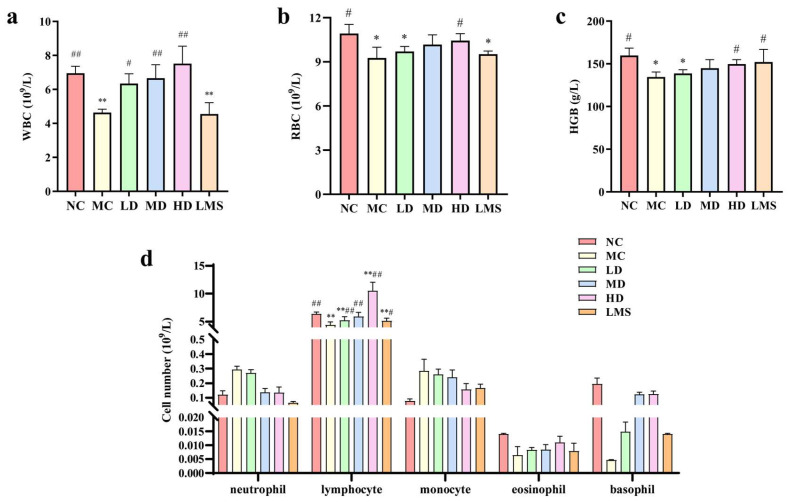
Effects of RPP on blood routine indexes of immunosuppressed *BALB/c* mice (n = 6). (**a**) WBC count; (**b**) RBC count; (**c**) Hb content; (**d**) the number and percentage of white blood cell types. Note: * *p* < 0.05, significantly different from the NC group; ** *p* < 0.01, extremely significantly different from the NC group; ^#^ *p* < 0.05, significantly different from the MC group; ^##^ *p* < 0.01, extremely significantly different from the MC group. Data are shown as mean ± SD.

WBC, RBC and Hb are readily accessible hematological parameters that provide supportive information on systemic inflammation and bone-marrow activity [27]. As shown in Figure 3a–c, the levels of WBC, RBC, and Hb in the MC group were extremely lower than those of the NC group (*p* < 0.01), indicating the successful establishment of an immunosuppressive model. Compared to the MC group, the counts of WBC, RBC, and Hb in RPP-treated groups were enhanced in a dose-dependent manner. Moreover, peripheral blood cells count in the LMS group were also improved compared to the MC group. These results demonstrated RPP robustly counteracted CTX-induced myelosuppression, accelerating the recovery of cells and thereby reinstating systemic immune competence in *BALB/c* mice.

Figure 3d showed that neither the MC group nor RPP-treated groups significantly altered the numbers of Granulocyte (neutrophil, eosinophil, and basophil) and monocyte compared with the NC group (*p* > 0.05). The MC group markedly reduced the lymphocyte count compared to the NC group (*p* < 0.01). Relative to the MC group, all RPP-treated groups significantly elevated the lymphocyte population (*p* < 0.05), indicating that RPP effectively restored lymphocyte counts in immunosuppressed *BALB/c* mice. Notably, the lymphocyte count in the MD group was indistinguishable from that in the NC group (*p* > 0.05). These results demonstrated that RPP robustly counteracted CTX-induced myelosuppression by accelerating the recovery of peripheral blood cells, thereby reinstating systemic immune competence in *BALB/c* mice.

### 2.5. Effects of RPP on Serum Immune-Related Indicators

**Figure 4:**
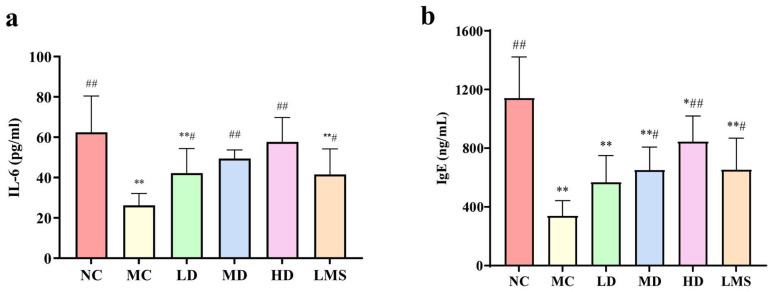
The immune-related indicators in the serum of immunosuppressed *BALB/c* mice (n = 6). (**a**) IL-6; (**b**) IgE. Note: * *p* < 0.05, significantly different from the NC group; ** *p* < 0.01, extremely significantly different from the NC group; ^#^ *p* < 0.05, significantly different from the MC group; ^##^ *p* < 0.01, extremely significantly different from the MC group. Data are shown as mean ± SD.

Interleukin-6 (IL-6) is a pleiotropic cytokine that governs B-cell maturation, acute-phase responses and the differentiation of T-helper cells, whereas immunoglobulin E (IgE) is the principal effector antibody of humoral immunity and an established surrogate of T helper 2 (Th2) immune response [28]. In CTX-induced immunosuppression, both mediators (IL-6 and IgE) undergo characteristically depletion compared with the normal state [29]. As shown in Figure 4a, the IL-6 level in the MC group dropped to 26.175 ng/mL, significantly lower than the NC group level of 62.424 ng/mL (*p* < 0.01). In contrast, RPP-treated groups and the LMS group exhibited markedly elevated IL-6 concentrations compared with the MC group (*p* < 0.05 or *p* < 0.01). Notably, IL-6 levels in MD and HD groups were restored to levels comparable to the NC group (*p* > 0.05), suggesting that RPP could enhance the secretion of IL-6 in immunosuppressed *BALB/c* mice.

Similarly, the concentration of IgE showed a strong correlation with the level of IL-6 (Figure 4b). The IgE level in the serum of the MC group showed a marked decrease versus the NC group (*p* < 0.01). CTX depressed serum IgE to 338.035 ng/mL, merely 29.6% of the NC level (1142.416 ng/mL). RPP administration restored IgE production in a dose-dependent manner, and MD and HD groups significantly elevated IgE levels compared with that of the MC group (*p* < 0.01). Notably, the HD group (844.852 ng/mL) reached about 70% of the NC level, which demonstrated a superior restoration of IgE relative to the LMS group (653.78 ng/mL).

### 2.6. Histological Examination of the Colon in BALB/c Mice

**Figure 5:**
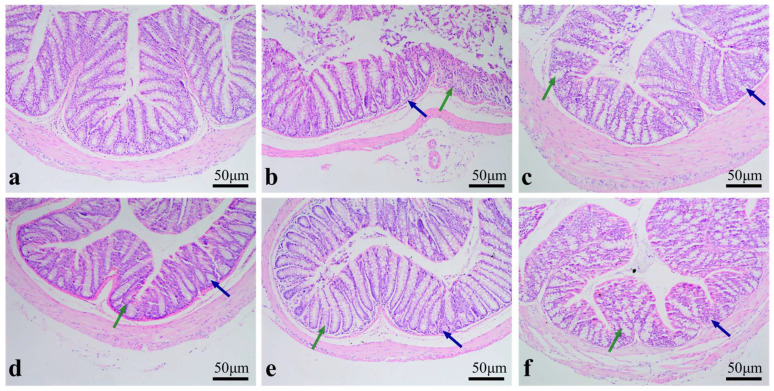
The effect of RPP on the colon morphology of immunosuppressed *BALB/c* mice (100 times) (n = 6). (**a**) NC group; (**b**) MC group; (**c**) LD group; (**d**) MD group; (**e**) HD group; (**f**) LMS group. Green and blue arrows indicated the status of goblet cells and crypts, respectively.

As shown in Figure 5b, the MC group exhibited sparse distribution, and fragmentation of goblet cells. As displayed in Figure 5c–e, the RPP-treated groups restored the colonic structure, which was characterized by clearly visible goblet cells, distinct crypts, and no observable infiltration of inflammatory cells. Among these groups, the HD group showed the most obvious recovery. These findings demonstrated RPP had the capacity to directly repair the intestinal damage caused by CTX and exerted a protective effect on the intestinal barrier.

### 2.7. Effects of RPP on Intestinal Tight Junction Protein Expression

**Figure 6:**
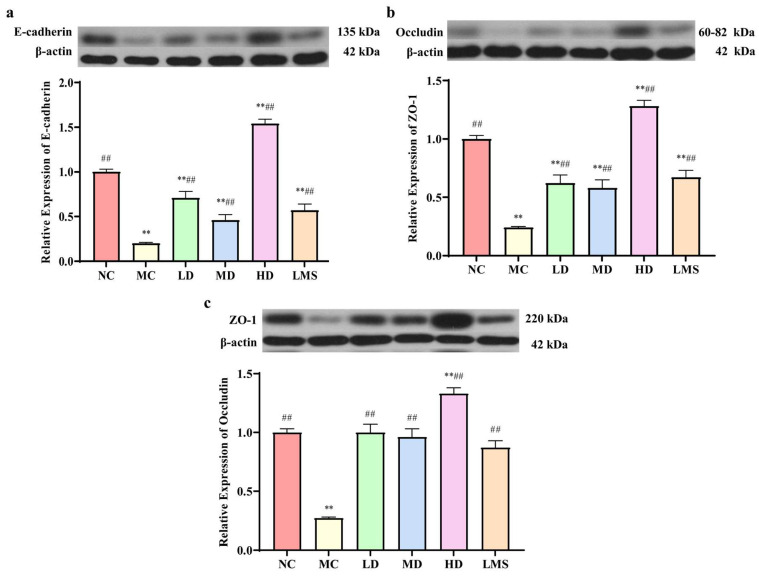
The effect of RPP on the expression of intestinal mucosal proteins in immunosuppressed *BALB/c* mice (n = 6). (**a**) The expression levels of the adherens junction protein E-cadherin; (**b**) The expression levels of the tight junction protein Occludin; (**c**) The expression levels of the tight junction protein ZO-1. Note: ** *p* < 0.01, extremely significantly different from the NC group; ^##^ *p* < 0.01, extremely significantly different from the MC group. Data are shown as mean ± SD.

The intestinal mucosal barrier is formed by epithelial cells that are closely joined by tight junction proteins, which are critical for maintaining intestinal homeostasis and ensuring barrier integrity and function [30]. To evaluate impacts of RPP on intestinal barrier integrity, the expression levels of tight junction proteins (ZO-1, occludin) and the expression level of the adherens junction protein (E-cadherin) in the colon were examined. As shown in Figure 6, compared to the NC group, the expression levels of E-cadherin, occludin, and ZO-1 were significantly decreased in the MC group (*p* < 0.01), indicating that CTX compromised the mechanical gut barrier. RPP treatment led to a markedly increased expression of junction proteins (E-cadherin, occludin, ZO-1) compared to the MC group, confirming its protective effect against CTX-induced intestinal barrier injury. Among these groups, the HD group showed the greatest improvement (*p* < 0.05 or *p* < 0.01). Similarly, compared to the model group, the LMS group exhibited a significant up-regulation of tight-junction proteins (*p* < 0.01). Furthermore, the HD group demonstrated markedly superior effects to those observed in the LMS group. These results indicated that RPP can enhance the intestinal barrier integrity in immunosuppressed *BALB/c* mice via upregulating the expression of tight junction proteins and consequently protecting the colonic barrier.

### 2.8. Effects of RPP on Intestinal Secretory IgA (sIgA) Levels

**Figure 7:**
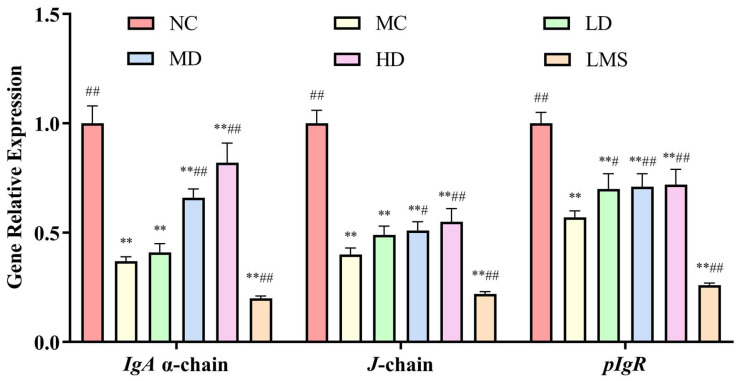
The effect of RPP on the expression of intestinal sIgA gene in immunosuppressed *BALB/c* mice (n = 6). RT-PCR data were normalized using the 2^−ΔΔCt^ method, with β-actin serving as the housekeeping gene. Note: ** *p* < 0.01, extremely significantly different from the NC group; ^#^ *p* < 0.05, significantly different from the MC group; ^##^ *p* < 0.01, extremely significantly different from the MC group. Data are shown as mean ± SD.

To further investigate the effects of RPP on intestinal immunity, this study employed Real Time-PCR (RT-PCR) to analyze the expression levels of the sIgA gene in the intestinal mucosa. SIgA, the predominant mucosa antibody, plays pivotal roles in gut immune homeostasis [31]. Its deficiency correlates with impaired mucosal immunity and excessive inflammation [32]. As shown in Figure 7, there was a significant reduction in *IgA* α-chain, *J*-chain and *pIgR* content in colon mucosa after CTX treatment relative to the NC group (*p* < 0.01), indicating that CTX can markedly affect sIgA component production. In comparison of the MC group, MD and HD groups showed markedly increased expression of *IgA* α-chain and *J*-chain (*p* < 0.05 or *p* < 0.01). Notably, the expression of *pIgR* gene was increased in all treatment groups relative to the MC group. The LD group showed a moderate improvement (*p* < 0.05), whereas the MD and HD groups demonstrated more pronounced enhancements (*p* < 0.01). Unfortunately, LMS failed to restore sIgA levels and caused a further numerical decrease.

## 3. Discussion

The immune system acts as a complex defense network that protects the body from harmful pathogens [1]. CTX is a commonly used clinical antitumor drug in practice, but it can cause significant immune dysfunction in the body [33]. Therefore, marine-derived polysaccharides have garnered attention for their potential immunomodulatory properties [13]. In this study, we investigated the effects of RPP on immune function in CTX-induced immunosuppressed *BALB/c* mice, aiming to explore the potential of RPP as a novel immunomodulator to mitigate CTX-induced immunosuppression.

CTX can cause weight loss and reduce food intake in mice, further leading to immunosuppression [34]. In our study, RPP treatment effectively mitigated these adverse effects, with high-dose RPP showing the most significant recovery in body weight and food intake [35]. This observation was consistent with previous studies on other marine-derived bioactivities, such as oyster polypeptides, red shrimp, and ulvan-type polysaccharides. They had demonstrated similar protective effects against CTX-induced weight loss and anorexia [19,36,37].

The spleen and thymus are key immune organs for cellular immunity and humoral immunity, and both are involved in the systemic adaptive immune process. Therefore, when seeking nutrients that enhance immunity, the spleen and thymus indices are usually regarded as preliminary indicators of immune health [34]. In this study, RPP significantly restored indices of the spleen and thymus, indicating its protective effects on immune organ integrity. Our result was corroborated by studies on OP-1 [14] and Enteromorpha polysaccharide (EP2) [24].

Hematological parameters, including WBC, RBC, and Hb, are critical indicators of systemic health and immune competence [38]. Our study found that RPP significantly elevated WBC, RBC, and Hb levels in a dose-dependent manner, with high-dose RPP showing the most pronounced effects. This finding was consistent with previous studies on other immunomodulators, such as High-molecular-weight fucoidan (Fucoidan P). It had also demonstrated the ability to restore hematological parameters in immunosuppressed mice [39]. The results of this study showed that RPP had similar immune repair effects to marine polysaccharides, suggesting that RPP can be used as a potential immune enhancer.

Immunoglobulins are key markers of humoral immunity and cytokines play vital roles in immune regulation. CTX-induced immunosuppression leads to significant reductions in the levels of key cytokines (IL-6) and immunoglobulins (IgE), which are essential for B-cell maturation and humoral immunity [40]. Our study found that RPP significantly upregulated the levels of IL-6 and IgE in a dose-dependent manner, with high-dose RPP achieving levels comparable to those in normal mice. The effects of RPP on restoring serum IL-6, IgE, and repairing immune organs may be mediated through the NF-κB and STAT3 pathways. The NF-κB pathway is activated via TLR4 to promote IL-6 transcription, while the STAT3 pathway is activated by IL-6 and further drives B cell differentiation into IgE-secreting plasma cells. This regulatory process is consistent with the known mechanisms of marine polysaccharide action [11,13,41]. However, whether this occurs via T follicular helper (Tfh) cell-mediated cognate interactions or through an intrinsic B-cell pathway remains to be elucidated [42]. Notably, RPP-induced IgE elevation should be viewed in the context of CTX-induced immunosuppression. IgE contributes to both allergy and physiological humoral defense, and CTX reduced IgE to 29.6% of the NC level. This reduction was a sign of impaired immunity rather than lower allergy risk [41]. RPP only restored IgE to approximately 70% of the NC level, with no abnormal overactivation. Moreover, no allergy symptoms or eosinophil aggregation were observed, as shown in Figure 5. Immunomodulator-mediated IgE recovery in such models supports protective immunity. This observation aligns with earlier reports on other immunomodulatory agents, such as *Cordyceps sinensis* spore polysaccharides [14] and *Schisandra chinensis* fruit polysaccharides [15]. The restoration of these immune mediators by RPP suggests its potential to enhance both cellular and humoral immunity. The Previous studies demonstrated that dandelion polysaccharides exerted anti-inflammatory effects. Their mechanism involved regulating protein expression through the IL-6/STAT3 pathway, which in turn contributed to mucosal tissue repair [43]. In our study, RPP was also able to regulate the expression level of IL-6; therefore, it is hypothesized that RPP effect on improving the immune function of immunosuppressed mice may be associated with the IL-6/STAT3 pathway.

The intestinal mucosal barrier serves as an essential immune safeguard, which is composed of intestinal epithelial cells and tight junction proteins. Intestinal permeability is associated with the general health status of the body. Goblet cells maintain the mucosal barrier by producing mucin, and villi length and crypt depth are directly related to intestinal integrity [44]. Our study found that CTX-induced immunosuppression leads to significant damage to the intestinal mucosal barrier, characterized by villus atrophy, goblet cell loss, and reduced tight junction protein expression. LMS is a classic immunomodulator. Its core mechanism of action is to activate central immune organs (such as the thymus and bone marrow), enhance the activity of immune cells, and regulate the secretion of cytokines and antibodies. Through these effects, LMS strengthens immune function and restores immune homeostasis. Notably, it does not act on the intestinal immune system. However, our study focused on the expression of sIgA in the colonic environment. This explaind why LMS failed to restore the sIgA level, as shown in Figure 6 [34,45]. While RPP significantly restored the intestinal mucosal barrier by upregulating the expression of tight junction proteins (ZO-1, occludin) and adherens junction protein (E-cadherin), as well as promoting the secretion of sIgA. These findings were consistent with previous studies on other marine-derived biological polysaccharides, such as fucoidan and ulvan-type polysaccharides [37,46]. Additionally, RPP-induced upregulation of intestinal tight junction proteins may depend on the AMPK pathway, as this pathway facilitates protein phosphorylation and membrane localization. Meanwhile, the enhanced secretion of sIgA might be associated with JAK/STAT6-mediated upregulation of *pIgR* expression [40,47]. The classical mechanisms of polysaccharide-mediated immune regulation provided a reasonable molecular explanation for the role of RPP in protecting the intestinal mucosal barrier and enhancing immune function. However, these hypotheses are based on the experimental data of this article and relevant literature. In future studies will be necessary to validate these pathways of RPP in greater depth at the molecular level.

## 4. Materials and Methods

### 4.1. Materials

RPP (yield: 30.54% (*w*/*w*), purity: 80.76% (*w*/*w*), was extracted by fermentation of a *Ruditapes philippinarum*-based medium inoculated with *Bacillus subtilis* natto BN-G (CCTCC M 2018080)) was provided by the Qingdao Engineering Research Center for Bioanalysis and Health Application (Qingdao, China). The molecular weight of RPP is 443.66 kDa. The monosaccharide composition (mol% of total sugars) was determined by PMP-HPLC: Glc, 99.74; Man, 0.26. RPP was a glucan with a backbone mainly composed of 1,4-α-D-Glc*p*, branched at the C-6 position. The molar ratio of 1,4,6-α-D-Glc*p* to 1,4-α-D-Glc*p* was approximately 1:4, with high branches. CTX was purchased from Baxter Oncology GmbH Co., Ltd. (Halle, Germany). Levamisole and Eosin Y were purchased from Shanghai Sangon Biotech Co., Ltd. (Shanghai, China). Mouse IL-6 enzyme-linked immunosorbent assay (ELISA) Kit (SXM032) was purchased from Shanghai Senxiong Technology Co., Ltd. (Shanghai, China). Mouse IgE ELISA Kit (ab157718) was purchased from Abcam Co., Ltd. (EGSC, Cambridge, UK). Xylene, was purchased from Aladdin Co., Ltd. (Shanghai, China). Anhydrous ethanol was purchased from National Pharmaceutical Co., Ltd. (Shanghai, China). Hematoxylin was purchased from Solarbio Co., Ltd. (Beijing, China). TRIpure and RNase inhibitor were purchased from Beijing BioTeke Co., Ltd. (Beijing, China). BCA Protein Assay Kit, SDS-PAGE Gel Rapid Preparation Kit, E-cadherin antibody (WL01482), Occludin antibody (WL01996), ZO-1 antibody (WL03419), Goat anti-rabbit IgG-HRP (WLA023), and internal reference antibody β-actin were purchased from Wanleibio Co., Ltd. (Shenyang, China).

### 4.2. Experimental Animal Grouping

Healthy SPF-level male *BALB/c* mice, aged 5 weeks and weighting 17–18 g, were purchased from Sibeifu Co., Ltd. (Beijing, China; No. SCXK [Jing] 2019-0010). Animal experiments were approved by the Animal Ethics Committee of the Medical College of Qingdao University (No. QDU-AEC-2023342) and conducted in accordance with the Guidelines for the Care and Use of Laboratory Animals of Qingdao University.

After 7 days of adaptive culture, 36 mice were randomly divided into six groups of 6 mice each [48,49]. These included a NC group, MC group, LD group, MD group, HD group, and LMS group. All groups except the NC group received intraperitoneal injections of 80 mg/kg CTX on days 1–3 to induce immunosuppression. The mice in the NC group were injected intraperitoneally with 100 μL of normal saline. From the fouth day, NC and MC mice were administered the same volume of normal saline via oral gavage, while LD, MD, and HD mice received RPP at doses of 500, 1000, and 2000 mg/kg/day, respectively, and the LMS group was given levamisole hydrochloride at 30 mg/kg/day [40] for 14 days. At the end of the intervention, mice were fasted for 12 h, before sample collection. Body weight was recorded, and blood samples were taken by retrobulbar puncture and then mice were euthanized by cervical dislocation. Liver, spleen, thymus, small intestine, and small intestine contents were then collected for further analysis.

### 4.3. Body Weight, Food Intake, and Organ Index

The body weight and general condition were recorded every 3 days. The spleens and thymuses were rinsed with normal saline, blotted dry, and weighed. Organ indices were calculated using the following formulae:

Organ Index (mg/g) = Organ weight (mg)/Animal body weight (g)(1)

### 4.4. Histopathological Observation

Macroscopic images of the primary lymphoid organs (thymus and spleen) were captured to document gross changes. A 1 cm colon segment was immersion-fixed in 4% neutral-buffered formalin. The remaining specimens were wrapped in foil, plunged into liquid nitrogen before transfer to −80 °C storage.

### 4.5. Hematological Analysis

A hematology analyzer (BC-5000Vet model from Mindray, Shenzhen, China) was used to measure blood counts from the blood samples collected in anticoagulant tubes. The analysis included WBC, RBC, Hb, neutrophils, lymphocytes, monocytes, eosinophils, and basophils.

### 4.6. Determination of IL-6 and IgE

Blood drawn into coagulation-activating tubes was left undisturbed for 30 min, then centrifuged at 4 °C, 4000 rpm, for 10 min and the supernatant was collected. IL-6 and IgE were detected using ELISA and performed in strict accordance with the kit instructions.

### 4.7. Hematoxylin and Eosin (H&E) Staining

The colon was fixed in a 4% paraformaldehyde solution for 24 h, dehydrated using xylene and anhydrous ethanol, embedded in paraffin, sectioned, stained with hematoxylin-eosin, and mounted with neutral resin. Histomorphological changes in the colon were observed under a light microscope.

### 4.8. Western Blot Analysis

Lysis buffer was added to the colon of each group and centrifuged at 12,000× *g* at 4 °C for 30 min. Proteins were detected using a BCA protein detection kit, isolated with 10% sodium dodecyl sulfate-polyacrylamide gel electrophoresis (SDS-PAGE), and transferred onto 0.45 μm polyvinylidene difluoride (PVDF) membranes. After blocking 5% nonfat dry milk, the membranes were incubated with primary antibodies (E-cadherin antibody diluted: Wanleibio, China, 1/1000; Occludin antibody: Wanleibio, China, 1/500; ZO-1 antibody: Wanleibio, China, 1/400; β-actin antibody: Wanleibio, China, 1/400) at 4 °C overnight. Subsequently, the membranes were washed four times for 8 min with 15 mL TBST, incubated with secondary antibody (Goat Anti-Rabbit IgG: wanleibio, China, 1/5000) conjugated with horseradish peroxidase for 2 h at room temperature and washed four times for 8 min with 15 mL TBST. ECL reagents were added for chemiluminescent imaging and band densities were determined by Image J software (version 1.41o). The ratio of proteins examined were normalized against β-actin.

### 4.9. RT-PCR Assay

Total RNA was isolated from approximately 0.1 g of colonic tissue. Each sample was placed in a sterile centrifuge tube, mixed with 1 mL TRIpure reagent, and homogenized in a chilled bead beater. After 5 min at room temperature, the lysate was centrifuged at 10,000× *g* for 10 min at 4 °C. The supernatant was transferred to a new tube, mixed with an equal volume of isopropanol, and stored at −20 °C overnight. The precipitated RNA was pelleted by centrifugation, washed once with 1 mL of 75% ethanol, briefly vortexed, and centrifuged again. The supernatant was discarded, and the pellet was air-dried, dissolved in 30 µL RNase-free water, incubated at room temperature for 2 min, and mixed thoroughly to obtain total colonic RNA. RNA concentration and purity were assessed with a small aliquot; the remaining RNA were stored at −80 °C until further analysis. Transcripts of the *IgA α*-chain, *J* chain and polymeric immunoglobulin receptor (*pIgR)*, which are essential for secretory IgA synthesis in the colonic wall, were quantified by reverse transcription followed by RT-PCR. The relative mRNA expression level of each sample target gene was calculated using 2^−ΔΔCt^ with β-actin as the reference gene. The cDNA synthesis was carried out with the reaction system outlined in Table 2. Target-gene mRNA sequences were retrieved from the National Center for Biotechnology Information (NCBI) database, and intron-spanning specific primers were designed in Table 3.

### 4.10. Statistical Analysis

Statistics were run in SPSS 26.0; data are mean ± standard deviation (SD). Comparisons among multiple groups were performed using one-way analysis of variance (ANOVA). Statistical significance was set at *p* < 0.05, and highly significant differences were defined as *p* < 0.01. Graphical representations were created with GraphPad Prism software (version 8.3).

## 5. Conclusions

The present study investigated the immunoenhancing effects of RPP on CTX-induced immunosuppressed *BALB/c* mice. The results demonstrated that RPP treatment significantly mitigated the weight loss and improved food intake caused by CTX. Furthermore, RPP exhibited no obvious toxicity to vital organs at the tested dosages, as evidenced by the organ indices assessment. And RPP treatment markedly elevated immune organ indices. This study confirmed that RPP safely restored systemic and mucosal immunity, as shown by increased circulating WBC, RBC, Hb, IgE and IL-6 together with elevated expression levels of E-cadherin, occludin, ZO-1, and sIgA. These results support further investigation of RPP as a potential immunomodulatory agent in functional products. Future studies should first verify key mechanistic pathways such as NF-κB, STAT3, AMPK, and JAK/STAT6 through experiments. On this basis, additional efforts should include combining 16S rRNA sequencing to clarify how RPP-induced microbiota changes interact with systemic immunity, pharmacokinetic profiling to guide formulation optimization.

## Figures and Tables

**Table 1:** Effects of RPP on the organ index of immunosuppressed *BALB/c* mice.

Groups	Vital Organ Index/(mg/g)			
	Liver	Kidney	Heart	Lung
NC	42.17 ± 0.21	15.25 ± 0.68	6.33 ± 0.21	7.24 ± 0.97
MC	46.97 ± 0.40	16.18 ± 0.89	6.40 ± 0.39	9.18 ± 0.30 *
LD	47.82 ± 0.78	16.56 ± 0.20	6.21 ± 0.10	8.86 ± 0.38
MD	43.01 ± 0.66	15.58 ± 0.63	6.93 ± 0.35	8.52 ± 1.21
HD	42.11 ± 0.72	15.52 ± 0.43	6.09 ± 0.58	8.14 ± 0.79
LMS	44.41 ± 0.37	14.44 ± 0.57	6.40 ± 0.34	8.02 ± 0.67

Note: Statistical analysis was performed using one-way analysis of variance (ANOVA). * Indicates a significant difference compared with the NC group (*p* < 0.05).

**Table 2:** RT-PCR reaction system.

Reagent	Volume (μL)
Cdna template	1
Upstream primer (10 μM)	0.5
Downstream primer (10 μM)	0.5
SYBR GREEN mastermix	10
ddH2O	8
Total Volume	20
Cdna template	1
Upstream primer (10 μM)	0.5

**Table 3:** Primer sequences for RT-PCR.

Name	Sequence	Primer Length	Tm (°C)
*IgA* α-chain F	TCTTGTCATACGCCTGTTT	19	50.9
*IgA* α-chain R	ATTAGGGTTCCTGCCATT	18	51.6
*J*-chain F	GTAACAGGTGACGACGAA	18	48.3
*J*-chain R	CTGGGTGGCAGTAACAAC	18	50.9
*pIgR* F	ACGCTCTTGGTAACTGTCT	19	49.1
*pIgR* R	TCTGCCTGAATACTCCTTG	19	50.4
β-actin F	CTGTGCCCATCTACCTCCAGCAGCAG	23	64.5
β-actin R	TTTGATGTCACGCACGATTTCC	22	63.2

Note: The gene for the *IgA* α-chain represents the constant region gene of the heavy chain of *IgA*, the *J*-chain refers to the gene encoding the Immunoglobulin Joining Chain, and the *pIgR* gene encodes the receptor for polymeric immunoglobulins. The primers were synthesized by GenScript Biotech Corporation.

## Data Availability

All data that support the findings of this available from the corresponding author on reasonable request.

## Abbreviations

The following abbreviations are used in this manuscript:

RPP	Fermented *Ruditapes philippinarum* polysaccharide
CTX	Cyclophosphamide
WBC	White blood cells
RBC	Red blood cells
Hb	Hemoglobin
IL-6	Interleukin-6
IgE	Immunoglobulin E
Th2	T helper 2
sIgA	Secretory IgA
OP-1	Oyster polysaccharides
IL-2	Interleukin-2
MCP	Microwave-extracted clam polysaccharide
ROS	Reactive oxygen species
T2DM	Type 2 diabetes mellitus
SCFAs	Short-chain fatty acids
MCFAs	Medium-chain fatty acids
SBA	Secondary bile acids
*R. philippinarum*	*Ruditapes philippinarum*
EP2	Enteromorpha polysaccharide
Fucoidan P	High-molecular-weight fucoidan
Tfh	T follicular helper
ELISA	Enzyme-linked immunosorbent assay
NC	Control
MC	Model
LD	Low-dose
MD	Medium-dose
HD	High-dose
LMS	Levamisole hydrochloride
H&E	Hematoxylin and Eosin
SDS-PAGE	Sulfate polyacrylamide gel electrophoresis
PVDF	Polyvinylidene fluoride
RT-PCR	Real Time-PCR
*pIgR*	Polymeric immunoglobulin receptor
NCBI	National Center for Biotechnology Information
ANOVA	One-way analysis of variance
